# Impact of Yoga Nidra on Heart Rate Variability in Coronary Artery Disease Patients Undergoing Coronary Artery Bypass Grafting: A Comparative Study

**DOI:** 10.7759/cureus.92668

**Published:** 2025-09-18

**Authors:** Hitha Antony, Amal Ashok, Sunil Chouhan, Ruchi Singh, Santosh Wakode, Danish Javed

**Affiliations:** 1 Physiology, All India Institute of Medical Sciences, Bhopal, Bhopal, IND; 2 Paediatric Surgery, All India Institute of Medical Sciences, Bhopal, Bhopal, IND; 3 Ayurveda, Yoga and Naturopathy, Unani, Siddha and Homoeopathy (AYUSH), All India Institute of Medical Sciences, Bhopal, Bhopal, IND

**Keywords:** autonomic nervous system, cabg, coronary artery bypass grafting, heart rate variability (hrv), parasympathetic activity, yoga nidra

## Abstract

Background

This study aimed to assess the changes in heart rate variability (HRV) before and after coronary artery bypass grafting (CABG) in patients with coronary artery disease (CAD) and evaluate the effect of Yoga Nidra as a postoperative intervention on autonomic function during recovery.

Methodology

A prospective study was conducted among 32 patients diagnosed with CAD and scheduled for elective CABG. Preoperative and postoperative HRV measurements were recorded. After discharge, participants were randomly divided into the following two groups: the case group received the Yoga Nidra intervention (guided sessions, two sessions daily for three months), while the control group received standard care without Yoga Nidra. HRV was assessed again three months postoperatively. Parameters such as high-frequency (HF) power, low-frequency (LF) power, and LF/HF ratio were analyzed to evaluate autonomic nervous system (ANS) modulation.

Results

In the case group, a significant decrease in HF (normalized units) was observed two weeks after surgery (from 40.38 to 30.85), reflecting a transient reduction in parasympathetic activity due to surgical stress. However, by the end of the three-month Yoga Nidra intervention, HF values improved significantly to 40.98, indicating a parasympathetic rebound. A decrease in the values of LF and the LF/HF ratio was noted, suggesting a restoration of autonomic balance in the Yoga Nidra group compared to the controls. In other words, at the end of three months, the HF component showed a statistically significant improvement in the Yoga Nidra group compared to controls (p = 0.003). The 95% confidence interval for the group difference (4.80, 21.50) did not cross zero, and the effect size (r = 0.61) indicated a large effect.

Conclusions

This study demonstrates that while CABG initially disrupts autonomic function, as evidenced by reduced parasympathetic markers, regular Yoga Nidra practice contributes to modulating the balance of the ANS during recovery periods, facilitating a favorable shift toward parasympathetic dominance. This shift, as assessed by HRV parameters, suggests improved autonomic recovery and enhanced myocardial electrical stability during the postoperative period.

## Introduction

Coronary artery disease (CAD) is a significant heart condition that affects people globally. It is the leading cause of death in both high-income and low-income countries. Several risk factors can lead to CAD, including diabetes, high blood pressure, smoking, high cholesterol, obesity, stress, unhealthy lifestyle, environmental issues, and genetics. CAD is usually treated with medications such as antiplatelet drugs, nitrates, beta-blockers, and calcium channel blockers, which help reduce chest pain (angina) and manage the condition [1]. CAD is the leading cause of death and disability worldwide, especially in low- and middle-income countries, causing around 7 million deaths and 129 million lost healthy years each year, which places a heavy economic burden globally [2].

Regarding the surgical management, coronary artery bypass graft (CABG) surgery is one of the most common surgeries performed worldwide. In three-vessel disease, which involves a narrowing of 50% or more in all three main coronary arteries, CABG provides patients with a seven-month survival advantage compared to medical treatment. Patients with left primary artery disease show the most significant improvement in survival. Those with three-vessel disease and those with one- or two-vessel disease follow this. Patients with abnormal exercise tests and poor left ventricular (LV) function live longer than those without these conditions. After CABG, the heart rate control of the autonomic nervous system (ANS) is disrupted. Studies have shown that heart rate variability (HRV) decreases, and there is no significant recovery within the first six weeks after surgery. The reasons for reduced HRV after acute myocardial infarction and CABG vary. HRV usually returns to its pre-surgery level within six months. The early drop in HRV seen after surgery may be due to the immediate effects of the operation. The recovery of HRV seen after six months, regardless of the patient’s condition before surgery, suggests that the ANS might have started to recover. Six months after surgery, the autonomic nerve injury recovers with an improvement in reperfusion, which improves almost all HRV indices compared with those preoperatively [3,4].

According to the Task Force of the European Society of Cardiology and the North American Society of Pacing, a significant relationship has been established between the ANS and cardiovascular mortality, including sudden cardiac death. One of the methods used to measure parasympathetic health is HRV. Studies have shown that depressed individuals, aging individuals, those working in constantly stressful conditions, and those with certain mood disorders experience reduced HRV. Changes in HRV show how the components of the ANS affect the heart. Measuring HRV is a simple, low-cost, and non-invasive way to check heart nerve function and predict heart problems, especially in individuals with coronary heart disease. Several studies have shown that HRV drops considerably after CABG. This drop is even greater than what happens after a heart attack. However, it remains unclear if lower HRV a few months after surgery affects the long-term outcome for these patients. The return of HRV in the second month after surgery, and its stability up to six months, may be due to better heart function, less heart muscle damage, and healing of the vagus nerve or sinus node. If the vagus nerve or sinus node heals fully, and blood flow to the heart improves after surgery, HRV may even be higher six months after surgery than it was before [5,6].

The most interesting and challenging research topics in recent times have been the mysteries of the human body. Apart from the scientists and researchers of the modern age, the ancient sages and seers had delved even deeper into the marvels and intricate workings of the inner realms of the living human organism. Indian Yogis have provided a few excellent techniques for the healthy functioning of all tissues and organs and a healthy mind. Yoga Nidra is one of them. Yoga Nidra (yoga = union or one-pointed awareness; nidra = sleep) or yogic sleep refers to a state of consciousness that occurs just before going to sleep. It is a form of guided meditation that has emerged in the literature in the last two decades as an intervention for various medical conditions, such as stress and mental health, and various clinical diseases. It is a non-invasive, cost-effective approach that is also easily accessible and can be performed in the privacy and comfort of the home. It has proven to be an effective and successful relaxation technique in various clinical settings. It combines guided mental imagery with a specific yoga posture called Shavasana (or “corpse pose”). Yoga Nidra aims to promote a profound state of relaxation, which differs from sleep as one is still aware of one’s surroundings. Yoga Nidra is increasingly recognized as a valuable practice for treating physical and mental illnesses.

HRV reflects the variation in time between heartbeats and serves as a key indicator of ANS function, balancing sympathetic and parasympathetic activity. Higher HRV is associated with better stress resilience and cardiovascular health, while lower HRV indicates physiological stress and autonomic dysfunction. Yoga Nidra practice stimulates parasympathetic activity, reduces stress hormones such as cortisol, and promotes physiological equilibrium. Research indicates that Yoga Nidra can lower heart rate and blood pressure, reduce sympathetic drive, increase vagal tone, and enhance HRV parameters, particularly high-frequency (HF) power, which suggests its potential in restoring autonomic balance. Linking HRV with Yoga Nidra offers a quantifiable, non-pharmacological approach to evaluating and supporting recovery, emotional regulation, and overall autonomic health in clinical settings. Many clinical studies have shown that Yoga Nidra meditation is associated with positive physiological changes, including improvements in several hematological variables, red blood cell counts, blood glucose levels, and hormonal status [7]. This study aims to investigate the HRV changes occurring in patients before and after CABG and assess the effect of the Yoga Nidra intervention on HRV in post-CABG patients.

## Materials and methods

Study design

This quasi-experimental study (non-equivalent, control group, pretest-posttest design) was performed jointly by the Department of Physiology, Cardiothoracic Vascular Surgery (CTVS), and Ayush at the All India Institute of Medical Sciences, Bhopal. The research was conducted over 1.5 years, from November 2022 to January 2024, followed by two months of data analysis.

Study participants

We included 32 patients with CAD scheduled for elective CABG surgery who needed a saphenous vein graft and agreed to participate in the study. The sample size was determined using G*Power software. CAD was diagnosed through invasive coronary angiography performed in the CTVS department. Referring to the findings of coronary angiography, the surgeon confirmed the diagnosis, and the patient was scheduled for CABG surgery. Following approval from the Institutional Ethics Committee, all patients scheduled for CABG surgery using a greater saphenous vein graft were invited to participate in the study. The surgeon’s assessment determined the choice of limb for vein harvest. During the preoperative visit, we obtained written informed consent from participants. Patients underwent standard preoperative blood tests, ECG, and echocardiography. If the tests were standard, the date for the surgery was assigned to them. Subsequently, they were taken to the Department of Physiology, where their medical history was recorded and a physical examination was performed. Afterward, the patients were subjected to preoperative HRV for baseline records. Then, on the assigned date, CABG was performed. After one week of surgery, patients were discharged. All patients were taken up for postoperative HRV two weeks after CABG to compare the pre- and postoperative values of HRV. Participants were randomly assigned to the Yoga Nidra and control groups using a computer-generated random number sequence. This method of allocation aimed to minimize selection bias and ensure that external factors did not influence group assignment. Patients were divided into two groups, namely, the case group and the control group. Using a random number generator software, CalcTape, 16 patients were assigned to the case group and 16 to the control group. The patients in the case group, after their discharge from the hospital, were subjected to Yoga Nidra sessions for three months. Two sessions of Yoga Nidra (in the form of an audio recording which a yoga expert prepared), which were 30-minute sessions, were performed daily in the morning and evening at home for three consecutive months. Patients in the control group did not listen to the Yoga Nidra sessions. All patients (both case and control groups) were assessed three months later to evaluate changes in HRV. Results were then compared and analyzed between the two groups.

Exclusion criteria

Participants were excluded from the study if they met any of the following conditions: (1) age under 18 or over 65 years to reduce age-related variability in HRV; (2) a known history of cardiovascular, neurological, or psychiatric disorders (e.g., arrhythmia, stroke, epilepsy, major depressive disorder) that could impact autonomic regulation; presence of comorbidities known to influence HRV, including uncontrolled diabetes mellitus (HbA1c >8.0%), hypertension not managed with stable treatment for at least three months, and thyroid disorders or other endocrine abnormalities; current use of medications that significantly alter autonomic function, such as beta-blockers, calcium channel blockers, antidepressants, and anticholinergics; and did not provide consent or required emergency CABG.

Ethical considerations

The study was conducted after receiving approval from the Institutional Human Ethics Committee at AIIMS, Bhopal (permission number: IHEC-PGR/2022/PG/Jan/33, dated September 12, 2022). Written informed consent was obtained from all participants before their inclusion.

Heart rate variability assessment

The autonomic evaluation was performed at room temperature in the morning to reduce the influence of diurnal fluctuations in HRV. Participants were told to have a light breakfast and abstain from caffeinated drinks, alcohol, smoking, and strenuous physical exercise for 12 hours before testing. A simple analogue amplifier was used to capture a five-minute ECG in supine position in lead II for short-term HRV analysis. The advice of the task force on HRV was followed. Power spectral analysis was performed. We used commercial software (Synescope 3.10, Sorin Group Company) to perform PSA using the fast Fourier transform. HRV recording was used for time domain analysis, such as standard deviation of the R-R intervals, standard deviation of the difference between adjacent R-R intervals, root mean square of the sum of squares of differences between adjacent R-R intervals, and number of R-R interval differences more than or equal to 50 ms.

The balance between sympathetic and vagal activity in the heart is reflected in the beat-to-beat variations of the cardiac cycle. This is typically assessed by analyzing fluctuations in the R-R intervals. Spectral analysis, usually done using fast Fourier transformation, breaks down the R-R intervals into specific frequency bands: high (0.15 to 0.40 Hz), low (0.04 to 0.15 Hz), very low (0.0033 to 0.04 Hz), and ultralow (below 0.0033 Hz). Parasympathetic tone is primarily reflected in the spectral analysis’s HF component. The sympathetic nervous systems influence the low-frequency (LF) component. The LF/HF ratio is considered a measure of sympathovagal balance and reflects sympathetic modulations. Recordings with excessive atrial or ventricular ectopic beats, as were those with other arrhythmias, were excluded from analysis [8-13].

Yoga Nidra procedure

Unlike other relaxation techniques, Yoga Nidra is easy to learn and does not require extensive training. In a clinical setting, Yoga Nidra was introduced to patients after CABG and continued at home for 12 weeks. Patients listened to a 30-minute guided recording twice daily, focusing on breath, body awareness, and visual imagery. The practice was conducted in a semi-dark room with patients lying in Shavasana, enhancing focus and balanced awareness. Before initiating the mental steps required to enter into the conscious sleep state of Yoga Nidra, the practitioner adopts the classic Shavasana or “corpse pose.” This key yoga posture involves facing the palms upward and ensuring that no parts of the body are touching each other. Initially, participants struggled to stay awake but adapted within five days.

Yoga Nidra was delivered in the following eight structured stages: (1) preparation - participants removed distractions and assumed a comfortable, relaxed position with eyes closed; (2) resolve (Sankalpa) - a personal, positive intention was mentally repeated; (3) rotation of consciousness - guided awareness moved systematically through body parts, following the brain’s motor map; (4) breath awareness - participants focus on taking 10 deep breaths, becoming mindful of their breathing pattern; (5) feelings and sensations - contrasting sensations (e.g., heaviness/lightness) were evoked to deepen awareness. The instructor encourages participants to experience contrasting physical sensations, such as feeling as light as a feather or as heavy as a rock; (6) visualization - guided imagery encouraged emotional exploration; (7) repetition of resolve - the original intention was reaffirmed; (8) closure - gentle movements were used to return to waking consciousness. The instructor helps participants gradually return to everyday awareness, guiding gentle body movements to reawaken the body and mind.

A certified instructor developed the recording to ensure practical guidance throughout the meditative process. This method offers a safe, structured relaxation intervention. Sessions lasted 30 minutes, one session was in the morning and one session in the evening, daily for 12 weeks. Participants followed audio instructions while lying in Shavasana, with adherence tracked through attendance records, WhatsApp check-ins, and weekly phone calls. The practice focused on cultivating awareness without any physical movement, progressively enhancing the depth of relaxation. After three months, HRV was assessed to evaluate the impact of Yoga Nidra on the ANS of the patients, showing its potential therapeutic benefits post-CABG surgery [14].

Sample size

The sample size was determined using G*Power software. Based on a previous related study, 25 participants were required to detect differences in various parameters before and after CABG as per the effect size achieved in previous related studies. Thus, we enrolled 32 participants in the current study, considering a potential 20% dropout rate. The effect size from the reference study (0.7624949) was utilized to determine our sample size [15].

Statistical analysis

Statistical analysis was conducted using SPSS Statistics Version 28 software (IBM Corp., Armonk, NY, USA). We used non-parametric statistical tests because the data did not follow a normal distribution.

## Results

A total of 32 participants were enrolled in the study. Participants’ baseline records were recorded for the different HRV parameters before the CABG. Two weeks after the CABG, patients were again subjected to postoperative assessment of HRV to compare the pre- and postoperative values. Patients were divided into two groups, i.e., case group and control group. The patients in the case group were subjected to Yoga Nidra sessions after discharge for three months. Patients in the control group did not listen to the Yoga Nidra sessions. All patients (both case and control groups) were assessed three months after surgery to determine the change in heart rate. Results were then compared and analyzed. After the baseline assessment, four patients passed away during the postoperative period and were therefore excluded from the postoperative study and data analysis. As a result, we analyzed the pre- and postoperative data of the remaining 28 patients.

The participants’ demographic details showed that the average age was 57.38 ± 7.88 years, the average height was 164.92 ± 6.92 cm, and the average weight was 66.31 ± 8.3 kg. For age, only one of the three tests (skewness, kurtosis, and Shapiro-Wilk) indicated normality, suggesting the data did not follow a normal (bell-shaped) distribution. All three tests showed normality for height, indicating the data were normally distributed. Similarly, weight data appeared normally distributed, as all three tests supported normality. Table 1 shows the changes in the HF component of HRV over time in two groups. Table 2 shows the changes in the LF/HF ratio of HRV over time in the two groups. Table 3 illustrates the changes in the LF component over time in the two groups. Each table represents the mean and standard deviation (SD) of HF, LF, and LF/HF of HRV in normalized units (n.u.) for both the case and control groups at three timepoints: preoperative, two weeks postoperative, and three months postoperative. The group difference and effect size are also mentioned in the respective tables. The Wilcoxon-Mann-Whitney test was used to compare the distributions between the groups at each time point. A p-value <0.05 was considered statistically significant.

**Table 1:** Changes in the HF component of HRV over time in the two groups. The Wilcoxon-Mann-Whitney test was used to compare the case and control groups. HRV = heart rate variability; HF = high-frequency power; SD = standard deviation; CI = confidence interval; n.u. = normalized units

HRV: HF (n.u.)	Case group, mean (SD)	Control group, mean (SD)	P-value	Group difference (95% CI)	Effect size (r)
PreOperative	40.38 (12.71)	31.49 (10.15)	0.058	-0.03, 18.50	0.36
2 weeks	30.85 (7.56)	27.36 (8.09)	0.131	-2.00, 9.50	0.27
3 months	40.98 (10.97)	27.77 (7.97)	0.003	4.80, 21.50	0.61

**Table 2:** Changes in the LF/HF ratio over time in the two groups. The Wilcoxon-Mann-Whitney test was used to compare the case and control groups. HRV = heart rate variability; HF = high-frequency power; LF = low-frequency power; SD = standard deviation; CI = confidence interval; n.u. = normalized units

HRV, LF/HF	Case group, mean (SD)	Control group, mean (SD)	P-value	Group difference (95% CI)	Effect size (r)
Preoperative	0.87 (0.61)	2.04 (1.32)	0.052	-2.22, 0.01	0.38
2 weeks	0.96 (1.17)	0.80 (0.91)	0.014	0.05, 1.30	0.47
3 months	0.21 (1.40)	1.84 (1.01)	0.052	-2.50, 0.01	0.40

**Table 3:** Changes in LF over time in two groups. The Wilcoxon-Mann-Whitney test was used to compare the case and control groups. HRV = heart rate variability; LF = low-frequency power; SD = standard deviation; CI = confidence interval; n.u. = normalized units

HRV, LF (n.u.)	Case group, mean (SD)	Case group, mean (SD)	P-value	Group difference (95% CI)	Effect size (r)
Preoperative	47.79 (11.43)	56.67 (5.44)	0.016	-15.5, -1.9	0.50
2 weeks	64.21 (12.02)	60.01 (15.33)	0.672	-6.1, 10.4	0.87
3 months	45.65 (19.17)	43.76 (14.53)	0.711	-9.8, 13.2	0.74

For the HRV parameter HF (n.u), although the preoperative comparison showed a p-value of 0.058 indicating a trend toward significance, the 95% confidence interval for the group difference (-0.03, 18.50) includes zero, reflecting uncertainty in the magnitude of the effect. The effect size (r = 0.36) suggests a moderate effect, underscoring potential clinical relevance despite the borderline p-value. At three months, the statistically significant difference (p = 0.003) between groups is supported by a confidence interval (4.80, 21.50) that does not cross zero, indicating a reliable and meaningful difference. The effect size of 0.61 further indicates a large effect, highlighting a substantial improvement in the case group compared to controls.

## Discussion

Understanding the balance within the ANS is essential, particularly because the parasympathetic branch exerts greater influence than the sympathetic branch. Therefore, for sympathetic activity to increase, parasympathetic tone must be reduced. A stronger parasympathetic tone is linked to higher HRV, which is thought to support a more electrically stable heart muscle. Conversely, electrical instability in the heart may develop when sympathetic activity prevails. A decrease in HRV typically indicates this instability.

An electrically unstable myocardium is more prone to abnormal electrical activity, which can lead to premature ventricular contractions, severe arrhythmias, or even sudden cardiac death. Given these potential outcomes, regulating sympathetic and parasympathetic systems has become a growing focus in cardiovascular research. This interest stems from the connection between reduced parasympathetic activity, sympathetic dominance, increased risk of cardiovascular disease (CVD), and higher mortality rates. Wolf et al. were the first to establish a connection between decreased HRV and increased mortality following a myocardial infarction, as early as 1978. By analyzing one-minute ECGs.

Individuals with greater sinus arrhythmia, reflecting higher variability in heart rhythms, had a lower risk of death compared to those with lower variability. Acute myocardial infarction typically causes a marked drop in HRV, primarily due to ischemia and partial damage to heart muscle tissue. Injured and non-contracting areas of the left ventricle activate sympathetic sensory and motor pathways, resulting in elevated sympathetic nervous system activity. This heightened activity leads to decreased HRV, increased electrical instability in the heart muscle, and a higher risk of potentially fatal arrhythmias. Moreover, excessive sympathetic stimulation can inhibit the vagus nerve’s (parasympathetic) influence on the sinus node, decreasing HRV. Low HRV signifies a reduced capacity of the heart to adapt to physiological demands. A persistently low HRV following CABG is particularly concerning when accompanied by a low ejection fraction. As HRV often correlates with ejection fraction, ongoing suppression of HRV after surgery may reflect deeper cardiac dysfunction. Nonetheless, the precise link between reduced HRV after CABG and long-term patient outcomes is still not fully understood, and further studies are needed to explore this relationship. Reduced parasympathetic activity contributes to CVD through multiple physiological pathways. These factors include a higher demand for oxygen by the heart, increased excitability of the ventricles, narrowing of the coronary arteries, reduced blood flow to the heart muscle (ischemia), rupture of arterial plaques, oxidation of low-density lipoprotein by macrophages, and a lowered threshold for triggering arrhythmias [16].

Consequently, interventions that lower sympathetic nervous system activity and/or enhance parasympathetic function may offer cardiovascular protection and support the prevention and management of CVD. A large body of research indicates that yoga practices can alleviate symptoms of stress, anxiety, and depression by improving ANS regulation, activating beneficial neurohormonal responses, and reducing sympathetic drive.

While ongoing studies continue to explore how yoga influences gene expression, current evidence suggests it may play a role in regulating genes linked to cardiovascular health. Different elements of yoga work through varied and complex physiological and psychological mechanisms, including physical postures (asanas), controlled breathing (pranayama), meditation, and Yoga Nidra. Among these, Yoga Nidra is particularly noted for its cardiovascular benefits, as it activates the body’s relaxation response, decreasing sympathetic activity and enhancing parasympathetic tone.

Yoga Nidra effectively encourages both bodily and mental relaxation through several mechanisms. Yoga Nidra also stimulates alpha brain wave activity, which is associated with a calm, relaxed mental state. As the mind and body are deeply interconnected, physical relaxation naturally produces a soothing effect on the nervous system. As the central nervous system calms, the ANS also moves toward balance, reducing overall physical and mental activity. This decrease in muscular and neural effort results in a lowered metabolic rate. The muscles soften in this deeply relaxed state, and vasodilation occurs, allowing blood vessels to expand. This leads to reduced cardiac output and less strain on the heart, reflected in lowered systolic and diastolic blood pressure and a slower pulse rate. Additionally, the body’s oxygen demand decreases, reducing the respiratory rate. Yoga Nidra may also serve as a promising preventive method against ischemic heart disease, often referred to as the silent killer. Because stress is implicated in numerous diseases, it is a priority to include a focus on stress management and the reduction of negative emotional states to reduce the burden of disease. Alpha ECG dominance has been found during the practice of Yoga Nidra, which is characterized by mental relaxation [17,18].

Dol [14] conducted a study involving 40 university students selected through convenience sampling in a non-equivalent control group pretest-posttest design. A total of 20 students were assigned to a Yoga Nidra intervention group, while the remaining 20 formed the control group. Participants’ levels of life stress and self-esteem were assessed. The findings revealed that the Yoga Nidra group experienced a significant reduction in perceived life stress compared to the control group and their self-esteem scores significantly improved compared to the control group. Their results suggest that Yoga Nidra can reduce stress and enhance university students’ self-esteem. The study supports the idea that Yoga Nidra may enhance parasympathetic nervous system activity through its relaxation effects, which, in turn, helps alleviate psychological and emotional stress. Prior research has also indicated that yoga nidra may influence psychoneurophysiological functions, including increased EEG activity and stimulating dopamine, imipramine, and other hormones related to mental health and emotional well-being.

Ahuja et al. conducted a systematic review and meta-analysis to assess the effectiveness of Yoga Nidra in managing hypertension. The study revealed that Yoga Nidra led to a significant reduction in both systolic and diastolic blood pressure compared to control groups. These results support the role of Yoga Nidra as a beneficial complementary therapy for hypertension. It is a safe, affordable, and easily accessible practice that induces deep relaxation and improves interoceptive awareness, helping to address key factors associated with high blood pressure, including stress, vascular inflammation, and increased peripheral resistance. The meta-analysis shows that Yoga Nidra can effectively lower blood pressure, highlighting its potential as an adjunct therapy alongside standard medical treatments. The impact of Yoga Nidra on hypertension is mainly due to its ability to trigger a relaxation response, which decreases sympathetic nervous system activity while boosting parasympathetic function. This relaxation effect primarily acts on the brain and calms the nervous system. By dampening sympathetic overactivity and helping regulate heart rate, Yoga Nidra reduces peripheral vascular resistance and blood pressure. Additionally, Yoga Nidra supports mental health by alleviating conditions such as anxiety, depression, and stress, which, in turn, can further lower blood pressure. Similar to meditation, Yoga Nidra may also reduce vascular inflammation, offering another pathway through which it positively influences blood pressure [19].

Swami Satyananda Saraswati (1975) describes Yoga Nidra as a transitional state between wakefulness and dreaming, where deeper layers of the mind are accessed. The practice enhances bodily awareness, stimulating brain function and promoting deep relaxation by systematically directing attention across body parts. This sequence mirrors the “motor homunculus” map in the brain, activating corresponding neural circuits and facilitating pranic energy flow.

A key feature of Yoga Nidra is the focus on contrasting sensations or emotions, aligning with natural brain rhythms and promoting alpha wave activity associated with mental calmness. This mind-body integration leads to reduced autonomic activity, decreasing muscular tension, neural stimulation, and metabolic rate, supporting overall nervous system relaxation [20].

The present study examined the effect of Yoga Nidra on HRV in patients after CABG surgery. When we compared the two groups, those who practiced Yoga Nidra (case group) and those who did not (control group), the results suggest that Yoga Nidra helped improve balance in the ANS after surgery. While both groups had stress after surgery, those practicing Yoga Nidra showed better recovery in HRV in the next three months. The yoga therapy program for three months has significantly improved HRV parameters, including increased HF and decreased LF and LF/HF ratio. Through deep relaxation and controlled breathing techniques, Yoga Nidra practices may activate the parasympathetic nervous system via the vagus nerve, leading to enhanced vagal tone and improved HRV. Additionally, it reduces sympathetic overactivity by modulating stress-related pathways, including the hypothalamic-pituitary-adrenal axis and the renin-angiotensin system.

The decision to employ Yoga Nidra as the intervention in this study was guided by its unique suitability for postoperative recovery in CAD patients undergoing CABG, particularly in modulating autonomic function. Unlike other relaxation or mindfulness-based techniques, Yoga Nidra is a guided, effortless meditative practice that allows patients to enter a deeply relaxed state while remaining awake and mentally alert. This is important in the early recovery phase following CABG, where there can be several limitations such as physical limitations, emotional distress, and pain which can restrict the ability to engage in more active or physically demanding forms of therapy, such as asanas, pranayama, or conventional exercise-based rehabilitation.

Our present study had certain limitations, including the exclusion of deceased patients. First, in this study, four patients expired during their postoperative period. Excluding those deceased patients limited the ability to study the total outcomes. Second, the small sample size limits the statistical power and generalizability of the findings. Third, the quasi-experimental design, lacking a placebo or active control group, introduces the possibility of bias and limits causal inference. Fourth, the short follow-up period may not fully capture the long-term effects or sustainability of the intervention. Fifth, the lack of formal adherence tracking to the Yoga Nidra practice may influence the reliability of observed effects. Additionally, potential confounding from concurrent medications affecting autonomic function was not fully controlled, which could have impacted HRV outcomes.

## Conclusions

HRV has emerged as a valuable non-invasive tool for monitoring autonomic function in cardiac patients. In this preliminary study, Yoga Nidra appeared to elicit favorable changes in HRV parameters among post-CABG patients, suggesting its potential as a simple, safe, and supportive strategy for autonomic recovery. These findings underscore the possible therapeutic role of yoga-based mind-body interventions in enhancing cardiovascular health and contributing to cardioprotective effects during the postoperative period. The study offers initial evidence of the dynamic interaction between psychological relaxation techniques and physiological responses, particularly in the context of ANS modulation. Despite being a meditative practice, Yoga Nidra is relatively easy to learn and implement, which may enhance its acceptability and adherence among cardiac patients during recovery. Larger, multicentric trials with longer-term follow-up and robust control conditions must confirm the observed benefits and clarify the underlying mechanisms. Future research should focus on addressing the limitations of this study through larger, randomized controlled trials with longer-term follow-up. Future research should aim to elucidate the specific neurophysiological and biochemical mechanisms by which Yoga Nidra modulates ANS activity. This includes exploring its impact on stress hormone levels, vagal tone, and brain-heart interactions using neuroimaging and biomarker analysis. Additionally, studies should assess the long-term durability of HRV improvements and their correlation with meaningful clinical outcomes such as reduced rehospitalization rates, improved functional capacity, and enhanced quality of life. From a clinical integration standpoint, Yoga Nidra holds promise as a feasible, low-cost, and non-invasive adjunct to conventional postoperative care for CABG patients. Its simplicity and minimal resource requirements make it well-suited for inclusion in cardiac rehabilitation programs, especially in resource-limited settings. Further investigation into implementation strategies, including patient education, digital delivery platforms, and integration with existing rehabilitation protocols, will be important to facilitate its adoption in routine clinical practice.
